# Mothers’ conceptions about excess weight in infancy and the nutritional status of their children

**DOI:** 10.6061/clinics/2016(09)03

**Published:** 2016-09

**Authors:** Janaína Paula Costa da Silva, Vicente Sarubbi Junior, Viviane Gabriela Nascimento, Ciro João Bertoli, Paulo Rogério Gallo, Claudio Leone

**Affiliations:** I Universidade de São Paulo, Departamento Saãde Materno-Infantil, São Paulo/SP, Brazil; II Faculdade de Ciências da Saúde do Trairi da Universidade Federal do Rio Grande do Norte/RN, BrazilBrazil; III Universidade de Taubatã, Departamento de Medicina, Taubaté/SP, Brazil

**Keywords:** Mothers, Preschool Children, Overweight, Obesity

## Abstract

**OBJECTIVE:**

To analyze maternal conceptions about excess weight in infancy and the nutritional status of their preschool-aged children.

**METHODS:**

A mixed, exploratory study was performed using semi-structured interviews. Two study groups were defined: a group of 16 mothers of children with excess weight and a group of 15 mothers of eutrophic children. The interviews were submitted to content analysis using CHIC software (*Classification Hiérarchique Implicative et Cohésitive*^®^).

**RESULTS:**

The mothers of children with excess weight tended to conceive thin children as malnourished, while those of normal weight children emphasized the influence of family and genetics as determinants of a child’s nutritional status. Although there was a certain consensus among the mothers that an unhealthy diet contributes to the risk of a child developing excess weight, the concept of genetics as a determinant of a child’s nutritional status was also present in the dialogue from the mothers of both groups. This result indicates a lack of clarity regarding the influence of eating behavior and family lifestyle on weight gain and the formation of a child’s eating habits. Both groups indicated that the mother has a decisive role in the eating habits of her child; however, the mothers of children with excess weight did not seem to take ownership of this concept when addressing the care of their own children.

**CONCLUSION:**

Differences in conceptions, including taking ownership of care, may contribute to the development of excess weight in preschool-aged children.

## INTRODUCTION

Excess weight in children, meaning overweight or obesity, has shown worrying trends in terms of prevalence 1 and prognosis 2 and has gained growing interest from health professionals in addition to society as a whole. It is believed that maternal factors related to pregnancy, intrauterine growth and development and other characteristics of a child during the first year of life also influence the development of excess weight 3,4. Excess weight is in turn associated with important comorbidities in adolescents 5 and young adults 6,7.

Although it is not possible to characterize the existence of a consensus concerning the risk or protective factors associated with excess weight, the most widely studied characteristics for explaining the increased prevalence of excess weight in childhood over recent years have been birth weight 8, duration of exclusive breastfeeding 9, nutritional status and education of the parents, socioeconomic status and other family characteristics 10,11, the perception of excessive weight in childhood and/or adolescence and the understanding of parents of the possible complications of this nutritional alteration 12,13. Recent studies 14,15 conducted to investigate the perceptions of parents regarding the weight of their children found that parents often do not realize that their children have excess weight or even underestimate the weight of their own children.

Because obesity represents a major public health problem and considering that any interventions proposed to prevent or control excess weight for children of a young age necessarily require interactions with families 16,17,18, particularly mothers, it is particularly important to study the conceptions of mothers 19 regarding the nutritional status of their children.

Inadequate conception or non-recognition of excessive weight and/or obesity in a child by their mother may result in habits and maternal behaviors that contribute to the development and/or maintenance of excessive weight in children 20. As such, this aspect should be considered and addressed with mothers when designing interventions 19, whether individual or collective, that can help reverse the increasing trend of excess weight in children.

The objective of this study was to analyze agreements and disagreements in maternal conceptions of the nutritional status of their children among mothers of children with and without excess weight.

## METHODS

The current study was a mixed exploratory, quali-quantitative study. From a representative sample of 391 children attending preschools in the city of Taubaté (São Paulo State) that was previously used for a nutritional evaluation study conducted between 2011 and 2012 (denominated here as the matrix research), 31 mothers of children from the preschools’ maternal I class were interviewed 21. Based on the data from the survey matrix, mothers of children who were born weighing between 2500 and 3999 g, whose age during an anthropometric evaluation was between three and four years and who did not have any diseases or deformities that would make it impossible to evaluate nutritional status or that could interfere in the maternal perception of nutritional status, were selected for the present study. From the total children in the sample, two groups were selected according to the cutoff points for the z-score of body mass index (BMI) that are recommended by the Ministry of Health-Brazil 22. Group 1 (G1) included children with no excess weight, defined as a BMI z-score greater than or equal to -2 and less than or equal to 1 (≥-2 and ≤+1). Group 2 (G2) included children with excess weight, defined as a BMI z-score greater than 2 (>+2).

Because this study was carried out using semi-structured interviews, it was determined that at least 15 mothers had to be interviewed from each group. Allowing for potential losses and refusals to participate, an initial list of 20 children per group who met the inclusion criteria was prepared. The researcher contacted the mothers in the two groups via telephone. For G1, the order of contact with the mothers was established according to BMI, which was considered with regard to the closeness to the median (BMI z-score=0). For G2 (overweight or obese), the telephone contact order for scheduling the interviews was made using descending BMI order, starting with the children with the highest BMI z-scores.

Next, the selected mothers who agreed to participate were individually interviewed using a semi-structured questionnaire prepared solely for this research. The survey was previously tested. At the end of this process, 15 mothers remained in G1 and 16 in G2. Among the mothers from the original search, only two refused to participate in the interviews, one from each group, and the others were not contacted because the total required for the scheduled interviews had already been reached.

The interviews were recorded and their content transcribed in full. For the qualitative analysis, the data were processed using content analysis 23, which consists of analyzing collected verbal material and categorizing it into thematic units according to the responses related to the issues. The thematic units were transformed into variables and processed using CHIC^®^ software (*Classification Hiérarchique Implicative et Cohésitive*) to generate similarity trees (multilevel cluster) based on indices of more than 0.50 and less than or equal to 1.00 (Tables 1 and 2).

The Research Ethics Committee of the Public Health School of the University of São Paulo approved this study (protocol number 2157/2010). All procedures followed the criteria established by Resolution no. 196/96 of the National Health Council, in force at the time.

## RESULTS

### Study Population

In G1 (mothers of children without excess weight), the age of the mothers ranged from 24 to 43 years. Regarding individual characteristics, 10 of the 15 mothers reported that they had completed at least 11 years of schooling, 11 lived with a partner, and 10 worked. With respect to income, five had incomes below 1.8 times the minimum wage (MW), four between 1.8 to 3.7 times the MW, four above this amount, and two were not able to supply the information. As for housing, five mothers said they lived in their own house, four in a rented house and the others were not able to supply the information. In G2 (mothers of children with excess weight), the age of the 16 mothers ranged from 21 to 34 years; 13 reported having studied for at least 11 years; 14 reported living with a partner; 11 worked outside the home; 12 had a household income in the range from 1.8 to 3.7 times the MW; and half lived in their own home.

### Mothers’ Conceptions of the Nutritional Status of their Children

When asked, using the search form matrix, “In your opinion, is anyone who lives in the child’s home "fat" (overweight)?”, seven mothers answered yes, but none of them cited the child in the study as someone who was overweight. This coincides with the results of Myers and Vargas 24, who showed that 35.5% of parents did not recognize that their two- to five-year-old children were already obese. From the content analysis of all the interviews, it was possible to identify 13 thematic units. In the hierarchical analysis of similarities, data were found to have significant consistency with a minimum index of association of 0.56 for G1 (Table 1), while in G2, the minimum similarity index obtained was 0.61 (Table 2).

The results indicated that, for the most part, the issues concerning maternal conceptions regarding their children’s nutritional status were present in both groups. The difference between the groups was high in terms of the associations between the thematics found and regarding the presence of maternal permissiveness, which appeared explicitly only in G2. This result is probably due to the family being considered as the most responsible entity for the formation of a child’s eating habits, considering that they share many conflicts and common beliefs regarding the performance of their role within the social context in which they were found, as noted in recent studies 25,26.

As a result, the importance of studying a family’s eating behavior, beliefs, habits and lifestyle has gained attention in international research. Recent studies have investigated in-depth representations of the intra-family context that guides the eating habits of children, confronting the numerous implications involved in the group, social and economic contexts in which they occur 26,27,28. In this sense, whether beliefs are shared between parents or the nearest caregivers or through incorporating information from the scientific community, conceptions regarding the predisposition towards obesity are quite widespread when assessing discourses given by families 25,29.

## DISCUSSION

In the present study, the idea of genetic influence determining the nutritional status of a child was confused with the importance that family itself has in terms of food habits and lifestyle by the mothers of both groups:

“What makes a child become chubby…I think it depends a little on what they eat at home. I do not know. If a child is already plump, they have genetics to be chubby.” (mother from G1)

“…she is kind of chubby because she was born a little chubby.” (mother from G2)

“Obese parents, whether they want to or not, end up feeding their children more because they eat in a certain way and end up passing this on.” (mother from G1)

“…comes a little from the father and mother. Everyone at home is very short and chubby…and his father’s side is skinny and short. So I think the child is fatter because of the father and mother.” (mother from G1)

“Some children are not because of their predisposition? Because a child is born with a tendency to put on weight.” (mother from G1)

It is worth emphasizing that, for at least two decades, obesity treatments for children have had high dropout rates, showing that children and teenagers usually follow the eating patterns of their parents. Thus, the parents’ eating patterns must be modified or managed in conjunction with the treatment of the children, as there is a high possibility of failure when family factors are ignored 30,31. Pizzinatto’s study 32 described the desire of parents to encourage their children to want to be what they expected of them and observed how obese children have serious difficulties in developing perceptions of their own physical needs and in forming their own personal identity.

At the end of the twentieth century, Fields 33 highlighted the influence of the family relationship on teaching a child their first eating habits. The author noted that parents who are anxious and unaware of other reasons for why a child is crying end up giving food in response to the crying. However, this crying may instead be an alert that a child is hot, tired or has another discomfort. Thus, giving food in response to crying may end up causing a child to begin associating frustration or discomfort with the intake of food.

Among parents, physical size is still one of the main markers used to refer to whether their child is thin or overweight. For some parents, the prominent presence of fat, or ‘being fat’, is an indicator of reduced physical health in their child 26,29. In the present study, the mothers from G1 relayed that physical size is very important when discussing a child’s excess weight and reported what they did to prevent their children from gaining excess weight, such as properly portioning food:

“…I think obese is when you are very overweight, isn’t it? Having a belly and these things…” (mother from G1)

“So, if we teach [children] to eat only junk food, [they become obese]. Thus, if we teach the child to have a healthy diet, she will be healthy, won’t she?” (mother from G1)

“There is a limit to everything, a time to eat, a time to have breakfast, a time for dinner. All these times are respected. For this reason, I think she’s not out of the [normal] weight [range].” (mother from G1)

Meanwhile, the G2 mothers did not discuss their own conduct or how they physically perceived their children. In fact, in this group, the conception of having excess weight was linked more to external elements. For instance, they referred to damage being caused to the child due to their own routine activities, i.e., obesity was as a problem for the individual, although this did appear to be related to recognition of the important role that a mother has in educating her child about eating:

“Ah, weight, let’s say this: The weight [should be good] for the child’s height. If the child is very small and chubby, it will start to harm the child.” (mother from G2)

“I think that is when you start to do harm to health, when you start having high cholesterol, when it begins to increase like this. I think, when your weight is disrupting your life.” (mother from G2)

“Obese? According to a story on television, obese children are eating too much, a lot of junk. The mother put them there. They do not have a limit. The mother does not give a limit to the child to stop and lets the child eat. Hence, they will get fatter and fatter, and this will generate problems.” (mother from G2)

These conceptions are similar to those reported in Jain et al.’s 34 study, which found that conceptions of excess weight in children differ between mothers and health professionals. Mothers generally consider being overweight a problem when it causes a limitation in physical activity for the child, whereas healthcare professionals base their opinion on growth charts – a common reference tool. A parent’s perceptions of their child’s physical appearance may be quite inadequate when the child or preadolescent is overweight, making it necessary to address several factors apart from these perceptions, such as habits, tastes, eating patterns and physical activity, to improve awareness for the parent 29,35. As noted in Carnell et al. 25 and Thomas et al.’s 26 studies, it is important to investigate the motivations that lead parents to adopt certain strategies to ensure their children have a healthy diet. The availability of parents to monitor the eating routines of their children, in addition to cultural and personal values and expectations they have about feeding their children, exert a strong influence on the way the parents guide and manage their children’s eating behaviors 25,27,36.

The conceptions held by the mothers from G1 regarding what leads a child to develop excess weight were more directed to their children’s eating behaviors. Overeating, not having set schedules for eating and eating sweets or unhealthy foods – "chocolate, iced biscuits, fast foods, candy, soda" – were the main factors associated with childhood excess weight, as presented in the following reports:

“They mainly leave the rice, beans and vegetables on the side and only eat the fattening things. Fat, too much fried food, too much pasta. I think these things make you fat. It is eating too many stuffed cookies. Cookies, bread.” (mother from G1)

“…eating what you shouldn’t, eating a packet of stuffed cookies and soon after eating another one.” (mother from G1)

“…I think it is exaggeration at eating times, isn’t it? This is something that is not very healthy (…) Things like snacks. And they become overweight because of this…” (mother from G1)

For the G2 mothers, the factors that lead to a child developing excess weight also appeared to be conceptions about overeating and snacking:

“I think children who are fatter eat a lot of food…eat too much.” (mother from G2)

“…it is overeating.” (mother from G2)

“Because, sometimes, the child is chubby because of eating too much.” (mother from G2)

However, the report of one G2 mother revealed how perceptions of the amount of food ingested by a child may be inaccurate, such as when states that a child may be thin due to poor diet or to an inability to manage their hunger or satiety sensations:

“…because my older child does not eat as much as JWS. He [skinny brother] eats too little. I have to stay on top of him, to check that he eats. Because if we leave him, he has a little game, plays around, does not eat. If he is distracted, he stays hungry and does not care. I think that children who are fatter don’t get very hungry; they feel a little hungry and start asking, "Mother, I want this, I want that.” (mother from G2)

The expectations and conceptions of the mothers in G1 and G2 regarding their responsibilities in keeping their children well fed and regarding how much they could trust their children’s signs related to hunger and satiety were divergent and even conflicting 28,37. The way that a mother perceives her child’s eating behavior is sometimes reflected in her own life story 27. For both groups in the present study, the maternal figure was conceived as having a fundamental role in shaping the eating habits of her child. The conception that mothers could contribute to excessive weight gain in their children when they are permissive to the wishes of their children also appeared. However, the mothers of children with excess weight (G2) did not seem to take ownership of this concept when it came to their own children. In their reports, they spoke in the third person. Even the three mothers of the 16 respondents in this group who mentioned their own excess weight when they talked about the excess weight of their children did not include any reflections on the implications of this fact as it related to the nutritional status of their children:

“The child says what she want[s] and the mother gives it. I think it should not be like this. The child saying, ‘Oh, mum. I want this. Oh, mum. I want that’. And, sometimes, the mother, who is used to eating a lot, passes this behavior onto their child, who also eats too much.” (mother from G2)

This non-ownership by the G2 mothers regarding how their eating habits impact the food routines of their children reveals a possible relevant factor for determining the nutritional status of a child who presents excess weight. This suggests that, for G2, the mothers’ behaviors towards their children probably do not reflect the statements made about their roles in the formation of their children’s eating habits. Johnson et al. 28 stressed the importance of working with mothers on their concerns and fears, reinforcing their confidence in the self-regulation of their children and favoring experiences that promote their autonomy and the need to establish strategies to make their children less resistant to consuming healthier foods.

The most recent studies addressing the perceptions of families regarding the eating habits of their children have noted the importance of interventions to promote family participation and the inclusion of other important social elements in creating awareness of dietary habits during childhood 26,38,39,40. As Laurent 29, Martinez et al. 36 and Towns and D’Auria 41 noted, it is necessary to develop methods to help parents gain understanding of how to manage the weight of their children. Parental lifestyles, the environment and cultural, ethnic and economic aspects are influences that need to be considered when developing effective interventions for children with excess weight because these factors interact and can provide clinically significant results.

Similarly, it is essential that healthcare teams support parents in developing an understanding of their perceptions and in encouraging them to reflect on their conflicts and expectations regarding the adequacy of an intervention. Reinforcements are also necessary in the form government policies in the social field for the promotion of healthy eating habits and to provide dialogue and recognition, in a broad sense, of the various cultural and intra-family factors that shape the views of parents and, consequently, guide their eating habits 26,28. It is worth noting that the terms “conceptions,” “beliefs” and “parental cognitions” have been referred to in different studies, and there is no consensus on their exact overlap 42; in the current work, we adopted the term “maternal conceptions.” As Borges and Solomon 42 cited, maternal conceptions can be modified and are not conclusive, static beliefs.

In conclusion, although a certain consensus was found among the mothers that an unhealthy diet contributes to the risk of a child developing excess weight, the role of genetics as a determinant of a child’s nutritional status was still frequently cited in the reports from the mothers of both groups. This suggests a lack of clarity regarding the influence of eating behavior and family lifestyle on weight gain and the formation of a child’s eating habits. Although the G1 mothers (children without excess weight) did think that genetics influence weight, they also stated other factors, such as eating habits and lifestyle, as determinants of the nutritional status of a child. Some mothers in this group also referred to their own role in feeding their children. These conceptions, combined with the fact that the children of the mothers in this group did not have diagnosed nutritional alterations, indicate that these mothers possibly took ownership of the discourse they produced and, at the same time, acted on it.

In the G2 mothers (children with excess weight), some results suggested a conflict of ideas that could eventually inhibit a mother from exercising her role as a "protector" by helping her child avoid gaining excess weight. The first such result involved statements surrounding the non-involvement of a child when discussing what would lead a child to become overweight. Second, some mothers indicated a dissociation from the eating habits that led their children to become overweight. Third, some mothers stated a lack of confidence in their child’s capacity to self-regulate hunger and satiety. Finally, some mothers stated that they engaged in permissive behavior towards their child’s desires and requests.

The potential limitations of the current study include the sample used and the adoption of an interview method that used a semi-structured script, with no recording of direct observations of routine maternal behavior. Thus, generalization of the current results is difficult. However, the need to more deeply explore the motivations that lead a mother to opt for certain food strategies with regard to her child if the child already presents excess weight is evident. Such exploration should be conducted by the health team that is involved in assisting a family. According to the authors, these motivations can be well characterized by analyzing the thematic units identified in the interviews, especially regarding the differences between the two groups.

Other studies of the presence of and discrepancies between views and feeding practices of mothers or other relatives of children with excess weight will reinforce the hypothesis that these concepts affect not only the perception of a child’s nutritional status but also how a family responds to guidelines from health professionals. Thus, professionals, including nutritionists, should consider the importance of family conceptions, especially maternal ones, when defining the likelihood of adherence to guidelines for the care of an overweight child.

## AUTHOR CONTRIBUTIONS

Silva JP was responsible for planning the research, collecting and analyzing the quantitative and qualitative data, discussing the results, and writing and submitting the manuscript. Sarubbi Jr V was responsible for analyzing the qualitative data, discussing the results, and writing the manuscript. Nascimento VG was responsible for collecting the quantitative data and discussing the results. Bertoli CJ was responsible for collecting the quantitative data and discussing the results. Gallo PR was responsible for discussing the results. Leone C was responsible for planning and supervising the research, analyzing the data, discussing the results and producing the final revisions of the manuscript.

## Figures and Tables

**Table 1:** Indices of similarity between the conceptions of the mothers in G1 according to analyses performed using CHIC^®^ software (Taubaté, SP. Brazil, 2012).

Thematic unit (analysis variable)	Similarity index
Influence of family environmental factors (food, lifestyle and physical inactivity).	0.73
Influence/genetic tendency.	
Maternal role in food education.	0.65
Increased physical size/weight above normal.	
Excessive eating or without defined schedules.	0.56
Eating sweets or unhealthy foods.	

**Table 2:** Similarity indices between the conceptions of the mothers in G2 according to analyses performed using CHIC^®^ software (Taubaté, SP. Brazil, 2012).

Thematic unit (analysis variable)	Similarity index
Maternal role in food education.	0.90
Excess weight and its harm to a child.	
Recognition that a child is overweight.	0.88
Maternal recognition of their own excess weight.	
Mother's permissiveness with regard to the will of her child.	0.85
Obesity-related factors not associated with a child.	
Association between being thin and having a poor diet or a child's inability to self-regulate.	0.77
Maternal role in food education.	
Excess weight and its harm to a child.	
Influence of family environmental factors (food, lifestyle and physical inactivity).	0.71
Influence/genetic tendency.	
Eating sweets or unhealthy foods.	0.62
Increased physical size/weight above normal.	
Excessive eating or without defined schedules.	0.61
Mother's permissiveness with regard to the will of her child.	
Obesity-related factors not associated with a child.	
